# Ag85-focused T-cell immune response controls *Mycobacterium avium* chronic infection

**DOI:** 10.1371/journal.pone.0193596

**Published:** 2018-03-02

**Authors:** Bruno Cerqueira-Rodrigues, Ana Mendes, Margarida Correia-Neves, Claudia Nobrega

**Affiliations:** 1 Life and Health Sciences Research Institute (ICVS), School of Medicine, University of Minho, Campus de Gualtar, Braga, Portugal; 2 ICVS/3B’s, PT Government Associate Laboratory, Braga/Guimarães, Portugal; Fundació Institut d’Investigació en Ciències de la Salut Germans Trias i Pujol, Universitat Autònoma de Barcelona, SPAIN

## Abstract

CD4^+^ T cells are essential players for the control of mycobacterial infections. Several mycobacterial antigens have been identified for eliciting a relevant CD4^+^ T cell mediated-immune response, and numerous studies explored this issue in the context of *Mycobacterium tuberculosis* infection. Antigen 85 (Ag85), a highly conserved protein across *Mycobacterium* species, is secreted at the early phase of *M*. *tuberculosis* infection leading to the proliferation of Ag85-specific CD4^+^ T cells. However, in the context of *Mycobacterium avium* infection, little is known about the expression of this antigen and the elicited immune response. In the current work, we investigated if a T cell receptor (TCR) repertoire mostly, but not exclusively, directed at Ag85 is sufficient to mount a protective immune response against *M*. *avium*. We show that P25 mice, whose majority of T cells express a transgenic TCR specific for Ag85, control *M*. *avium* infection at the same level as wild type (WT) mice up to 20 weeks post-infection (wpi). During *M*. *avium* infection, Ag85 antigen is easily detected in the liver of 20 wpi mice by immunohistochemistry. In spite of the propensity of P25 CD4^+^ T cells to produce higher amounts of interferon-gamma (IFNγ) upon *ex vivo* stimulation, no differences in serum IFNγ levels are detected in P25 compared to WT mice, nor enhanced immunopathology is detected in P25 mice. These results indicate that a T cell response dominated by Ag85-specific T cells is appropriate to control *M*. *avium* infection with no signs of immunopathology.

## Introduction

The immune response to mycobacteria is a very complex process. Though it is known that several cell populations play important parts, from neutrophils, to macrophages (Mϕ), dendritic cells and CD4^+^ and CD8^+^ T cells, among others, the precise profile of a protective immune response against pathogenic mycobacteria is still unclear. CD4^+^ T cells have been shown to be absolutely required as they participate in the activation of antigen presenting cells and CD8^+^ T cells. However, the precise antigenic specificity required for an initial and prolonged protection is still debatable. A few reports showed CD4^+^ and CD8^+^ T cell expansions during the immune response to *Mycobacterium tuberculosis* [1–5] and these skewed TCR repertoires have been associated with more severe manifestations of disease [1,4], though a cause or consequence relationship remains to be established.

Several mycobacterial antigens have been shown to elicit a protective immune response such as ESAT-6, CFP10 or Ag85 [6]. Amongst these, the Ag85, present in three distinct variants (Ag85A, B or C), is one of the most well described antigens, being highly conserved across the *Mycobacterium* genus [7]. Ag85 is associated with the cell wall and is a major secreted protein [7,8] that has been successfully detected in the sputum of patients with pulmonary tuberculosis and in the cerebral spinal fluid of patients with meningitis tuberculosis [9]. *In vitro M*. *tuberculosis* infection of human monocytes and pulmonary Mϕ shown that the gene encoding for Ag85B, the *fbp*B, is expressed as soon as 4 h following infection, maintained for up to 5 days post-infection (dpi) [10,11]. In mouse models, Rogerson and colleagues showed that *fbp*B is expressed both in the acute and chronic phases of infection, peaking around 20 dpi [12]. More recently, proliferation of adoptively transferred CFSE-labelled Ag85B-specific CD4^+^ T cells was shown as soon as 14 days after *M*. *tuberculosis* infection [13,14] though this phenotype is lost when these cells are transferred into mice with over 4 wpi [14]. Altogether, these data supports the long accepted concept that Ag85 is an early-secreted protein during *M*. *tuberculosis* infection.

A specific Ag85 amino acid (aa) sequence, the peptide 25 (P25; aa240-254), has been shown to be the major mediator of CD4^+^ T cell cytokine production and proliferation in response to Ag85 [15]. In line with this, Takatsu and colleagues generated the P25 mouse model, whose T cells express a Vα5^+^Vβ11^+^ transgenic TCR that recognizes Ag85’s P25 in the context of I-A^b^ [16]. Upon *in vivo M*. *tuberculosis* infection, P25 mice presented an identical bacterial burden when compared with their WT peers at 4 wpi [17] suggesting that an early immune response to *M*. *tuberculosis* mostly directed to Ag85 is sufficient to control the bacterial burden to levels similar to the ones in WT mice with a diverse TCR repertoire.

To our knowledge, seldom work has been described on the relevance of Ag85 in the context of *M*. *avium* infection [8,18]. We investigated whether an immune response mostly directed at Ag85 is able to induce protection against *M*. *avium* infection and whether this response is enough to maintain long term protection. To do so, we have infected the P25 TCR transgenic mouse model, in a RAG sufficient background, with *M*. *avium* and evaluated their immune response in comparison to WT mice. Additionally, we assessed which T cells were more responsive to the infection, the Ag85-specific or the non-specific CD4^+^ T cells. Our results indicate that a T cell response mostly directed towards Ag85 is appropriate to control *M*. *avium* infection, at both initial and more prolonged phases, with no signs of immunopathology, despite the dominance of activated Ag85-specific T cells.

## Material and methods

### Mice

Breeding couples of WT (C57BL/6J) and TCRαKO mice (B6.129S2-Tcra^tm1Mom^/J) [19] were purchased from Charles River Laboratories (Barcelona, Spain) and from The Jackson Laboratory (Bar Harbor, ME, USA), respectively. Breeding pairs from P25 [C57BL/6-Tg(H2-K^b^-Tcra,-Tcrb)P25Ktk/J] [16] and Limited mice [20] were kindly provided by Dr. Anne O’Garra (The Francis Crick Institute, London, UK) and by Dr. Diane Mathis and Dr. Christophe Benoist (Division of Immunology, Department of Microbiology and Immunobiology, Harvard Medical School, Boston MA, USA), respectively. All animals were bred at the local animal facility and housed in a biosafety level 2 facility under pathogen-free conditions with sterilized food, water, bedding and environmental enrichment. Mice were 8 to 12 weeks old at the start of the experiments, which were performed in accordance with the EU Directive 2010/63/EU and the Portuguese legislation Decree-law 113/2013, approved by the local Animal Ethics Committee (*Comissão de Ética Animal conjunta* IBMC/INEB e ICVS). All personnel involved in the procedures were approved as competent for animal experimentation by the national competent authority for animal protection (*Direção Geral de Alimentação e Veterinária*, DGAV).

### Experimental infection and bacterial quantification

Infection with *M*. *avium* strain 2447 (provided by Dr. Françoise Portaels, Institute of Tropical Medicine, Antwerp, Belgium) was performed intravenously through the lateral tail vein, delivering 10^6^ colony forming units (CFU) per mouse, in a 200 μL volume of sterile saline with 0.05% of Tween80. At selected time-points post-infection, mice were euthanized by carbon dioxide inhalation; organs were aseptically removed and individually homogenized in sterile water with 0.05% of Tween80. Ten-fold serial dilutions of organ homogenates were plated onto OADC-supplemented Middlebrook 7H10 agar plates (Becton Dickinson, NJ, USA) and incubated at 37 °C for 7 days before bacterial burden determination.

### Spleen single-cell suspension preparation, ex vivo stimulation and IFNγ quantification by ELISA

Spleen single-cell suspensions were prepared by gentle disruption of the organs between two notched slide glasses. Erythrocytes were lysed using a hemolytic solution (155 mM NH_4_Cl, 10 mM KHCO_3_, 0.1 mM Na_2_EDTA, pH 7.2). After washing, cells were resuspended in supplemented RPMI 1640 (10% heat-inactivated fetal bovine serum, 10 mM HEPES, 1 mM sodium pyruvate, 2 mM L-glutamine, 50 mg/mL streptomycin and 50 U/mL penicillin, all from Merck, NJ, USA) and enumerated using a hemocytometer and 4% trypan blue.

For *ex vivo* stimulation, 5×10^5^ splenocytes were plated per well in a 96-well plate and incubated, in triplicate, in the presence of 4 μg/mL of Ag85_240–254_ peptide (*M*. *avium* sequence: FQDAYNGAGGHNAVF; Metabion, Germany). Incubation in the presence of 4 μg/mL of concanavalin A (ConcA) or in the absence of stimuli was used as positive and negative controls, respectively. Supernatants were collected 72 h after culture in a 37 °C, 5% CO_2_ incubator and IFNγ concentration was determined by ELISA [R4-6A2 and AN18 were used as capture and detection antibodies, respectively (eBioscience, Österreich, Austria)].

### In vitro T cell protection assay

Bone marrow (BM) derived Mϕ (BM-Mϕ) from WT mice were prepared from single-cell suspensions of BM cells aseptically flushed from femurs using a 25 G needle and syringe loaded with ice-cold supplemented RPMI (without penicillin and streptomycin). Cells were supplemented with murine Mϕ colony-stimulating factor (M-CSF; Peprotech, NJ, USA) to a final concentration of 20 ng/mL, and 5×10^4^ cells were plated per well of a 96-well plate. Cells were placed in the incubator at 37 °C and 5% CO_2_; 4 days later, 20 μL of M-CSF at 20 ng/mL was added per well. At day 7 of differentiation, media was replaced by 40 μL of *M*. *avium* inoculum at 2.5×10^6^ CFUs/mL and cells incubated for 4 h. Afterwards, BM-Mϕ were thoroughly washed with warm Hank’s Balanced Salt Solution (HBSS, Merck, NJ, USA) and left in supplemented RPMI (without penicillin and streptomycin) in the incubator. One day after infection, 1×10^5^, 5×10^4^ or 2.5×10^4^ purified CD4^+^ T cells from each mouse were added to the *M*. *avium*-infected BM-DMϕ. Briefly, CD4^+^ T cells were obtained from spleen single-cell suspensions (as described above) upon purification by negative separation using the CD4^+^ T Cell Isolation Kit and an autoMACS separator (both from Miltenyi Biotec, Germany), accordingly to the manufacturer’s instructions; a purity of 91±6% was achieved. All conditions were performed in triplicate; a set of wells was left with infected BM-DMϕ and without T cells. Viable bacteria quantification was performed 3 days later upon cells’ disruption with sterile H_2_O with 0.05% Tween80 and 0.5% saponin. Bacterial burden was also assessed in triplicate wells with purified T cells only (1×10^5^ T cells; no BM-DMϕ) representing less than 1% of the bacterial load of the respective T cells co-cultured with infected BM-Mϕ.

### Cell staining and flow cytometry

Cell surface staining was performed using specific antibody combinations directed to mouse CD3 (145-2C11), CD4 (RM4-5), CD8 (53–6.7), CD44 (IM7), CD62L (MEL-14), Vα2 (B20.1), Vα3.2b/c (RR3-16), Vα8.3 (B21.14), Vα11.1/.2 (RR8-1), Vβ5 (MR9-4) and/or Vβ11 (KT11; all purchased from BioLegend, CA, USA). Staining was performed on ice for 20 min using FACS buffer (PBS with 0.3% BSA, 0.01% sodium azide and 2 mM Na_2_EDTA).

For the tetramer stain, Ag85_240–254_-loaded I-Ab tetramer (*M*. *tuberculosis* peptide sequence: FQDAYNAAGGHNAVF; National Institute of Health Tetramer Core Facility, Emory University Vaccine Center, Atlanta, GA) was used and staining was performed for 1 h at 37 °C. Cells were thoroughly washed and stained with the surface antibody mix as described above.

For intracellular cytokine staining, 2×10^6^ cells were incubated with 16 μg/mL *M*. *avium* Ag85_240–254_ peptide or with a combination of 0.05 μg/mL Phorbol myristate acetate (PMA) and 0.5 μg/mL ionomycin, as a positive control. All conditions, including the non-stimulated control, were performed in the presence of recombinant murine interleukin 2 (IL2) at 100 U/mL. After 1 h of incubation (37 °C, 5% CO_2_), brefeldin was added to a final concentration of 10 μg/mL. Cells were further incubated for 5 h, upon which were washed and surface stained with antibodies directed to mouse CD3, CD4, CD8, Vβ11, Vα2, Vα3.2b, Vα8.3 and Vα11.1/2 (same clones as above). Cells were fixed and permeabilized with 2% paraformaldehyde and FACS buffer supplemented with 0.5% saponin, respectively, and intracellularly stained using antibodies directed to mouse IFNγ (XMG1.2), IL2 (JES6-5H4) and tumor necrosis factor (TNF; MP6-XT22; all from BioLegend).

Cells were acquired on a 12-color LSRII flow cytometer using FACSDiva software (Becton Dickinson, Franklin Lakes, NJ); data analysis was performed using FlowJo software (Tree Star, Ashland, OR). Gating strategies are represented in S1 Fig.

### Immunohistochemistry, immunofluorescence and liver histology

Detection of Ag85 and inducible nitric oxide synthase (iNOS) in tissues was performed by immunohistochemistry and immunofluorescence, respectively, in paraformaldehyde-fixed, paraffin-embedded tissues. Liver sections were briefly placed at 70 °C for paraffin melting and then rehydrated. Antigen retrieval was completed upon slide incubation with citrate buffer (pH 6; Sigma-Aldrich, Germany) at 96 °C, in a water bath, for 30 min followed by 20 min at room temperature. For iNOS, non-specific binding was blocked using 3% BSA in PBS with 0.05% Tween20 for 2 h at room temperature. Tissues were incubated overnight, at 4 °C, with a purified rabbit anti-mouse iNOS antibody (clone M-19; Santa Cruz Biotechnology, CA, USA). Detection was achieved using a goat anti-rabbit Alexa Fluor 488 (ThermoFisher Scientific, IL, USA) and nuclei were stained using Vectashield with 1 ug/mL of DAPI (Vector Labs, CA, USA). No significant signal is detected in liver sections from iNOS KO mice (data not shown). Quantification of iNOS^+^ lesions was performed from one liver section per mouse; five random fields were photographed under the 20× objective, one operator performed lesion classification blindly and the percentage of iNOS^+^ lesions was calculated per mouse. Ag85 staining was performed using the ImmPRESS^™^ Excel Amplified HRP Polymer Staining Kit (Vector Labs, CA, USA) following manufactures’ instructions, and the rabbit polyclonal antibody to *M*. *tuberculosis* Ag85B (Abcam, UK), which was incubated for 1 h at room temperature. No significant signal is detected in liver sections from non-infected mice or from mice infected with an Ag85KO-*M*. *tuberculosis* strain (data not shown). Upon Ag85 stain, bacilli were detected using the standard Ziehl-Neelsen procedure.

Determinations of lesion type (well-organized granulomas, poorly-organized granulomas and inflammatory infiltrates) in the liver were performed in hematoxylin and eosin stained sections. From one liver section per mouse, five random fields were photographed under the 20× objective, and lesion classification was performed blindly by two independent operators. The percentage of each lesion type was calculated per mouse.

All slides were visualized using a BX61 microscope with an Olympus DP70 camera. Image and area analysis was performed using Image J software (National Institutes of Health, MD, USA).

### Statistical analysis

**Fig 1:**
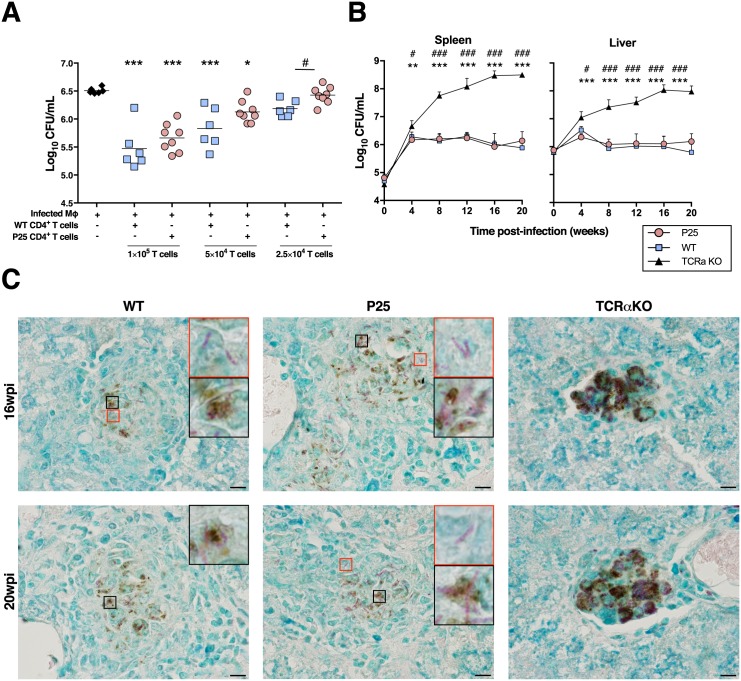
P25 mice control *M*. *avium* growth to the same extent as WT mice. **A**) CFU quantification on infected Mϕ, co-cultured with decreasing numbers CD4^+^ T cells isolated from WT or P25 mice at 4 wpi. * *p*<0.05, **p<0.005 and ***p<0.001 by one-way ANOVA test followed by Bonferroni post-hoc tests for the comparison of all groups to infected Mϕ, as a control; Student *t*-tests were performed for comparisons between the same amount of WT and P25 CD4^+^ T cells and represented by # for *p*<0.05. Depicted is one out of two independent experiments. **B**) Spleen (left) and liver (right) CFUs of WT, P25 and TCRα KO mice. Comparisons were performed by a two-way ANOVA test followed by Bonferroni post-hoc tests and differences marked with * are for comparisons of P25 *vs*. TCRα KO and with # for WT *vs*. TCRα KO; no significant differences are detected for the comparison of P25 *vs*. WT. * or # *p*<0.05, ** or ## *p*<0.005 and *** or ### *p*<0.001. Depicted is one (with 4 to 9 mice per group) out of two independent experiments. **C**) Representative liver sections stained for Ag58 (brown) of 16 and 20 wpi mice. Bacilli were detected by Ziehl-Neelsen staining (red). Scale bar, 10 μm.

Prism software (GraphPad v.7.a) was used for all the statistical analysis. Shapiro-Wilk normality test was used to assess the normal distribution of the data. As all data followed a normal distribution, parametric tests were performed: Student *t*-test to compare two groups; one-way analysis of variance (ANOVA) test, followed by Bonferroni post-hoc tests, to compare three or more groups; and two-way ANOVA, followed by Bonferroni post-hoc tests, to address strain and time of infection contribution to the evaluated variables. Differences were considered statistically significant for *p*-value <0.05. In the depicted graphs, symbols represent a single mouse and the horizontal line the mean, except in Fig 1B where each symbol represents the average of the group and the error bars the standard deviation. For some variables and in some instances, fold-variation was calculated as the average of the ratio of the non-infected mice (average of the group) over infected mice (each mouse individually).

## Results

### *M*. *avium* growth is controlled in P25 mice to levels similar to WT mice at early and late phases of the infection

Ag85_240-254_ from *M*. *tuberculosis* and *M*. *avium* differ in a single aa (A246G) located at one of the five peptide’s TCR recognition sites [21]. To determine the ability of P25 transgenic T cells to recognize *M*. *avium*-infected cells, an *in vitro* experiment was performed. When co-cultured with *M*. *avium*-infected BM-Mϕ, purified CD4^+^ T cells from 4 week *M*. *avium*-infected P25 mice control bacterial growth to similar levels when compared to WT CD4^+^ T cells (Fig 1A). Reduced protection conferred by P25 CD4^+^ T cells in comparison to WT is only detected when a very low number of CD4^+^ T cells are used (2.5×10^4^ T cells/well; which corresponds to a ratio of BM-Mϕ to T cells of 2:1; Fig 1A). Upon *in vivo* infection, P25 and WT mice present similar bacterial burden up to 20 wpi; TCRα KO mice (deprived from αβ T cells) and Limited mice (an alternative TCR transgenic model with diversity restricted at the CDR3 of the TCRα chain [20]) were used confirming that mice with no T cells, or with non-specific T cells, respectively, are unable to halt *M*. *avium* growth (Fig 1B and S2 Fig).

The ability of P25 mice to confer protection to similar levels as WT mice, up to 20 wpi, was surprising taking into consideration that Ag85 has been described as an early secreted antigen during *M*. *tuberculosis* infection [12,14]. However, in animals infected with *M*. *avium*, Ag85 is clearly detected, by immunohistochemistry, in the liver of WT, P25 and TCRα KO mice at late periods of infection (16 and 20 wpi; Fig 1C) revealing a continuous production of this protein by *M*. *avium* during chronic infection. Ag85 is detected within most, though not all, infected cells (Fig 1C, black and red insets, respectively).

**Fig 2:**
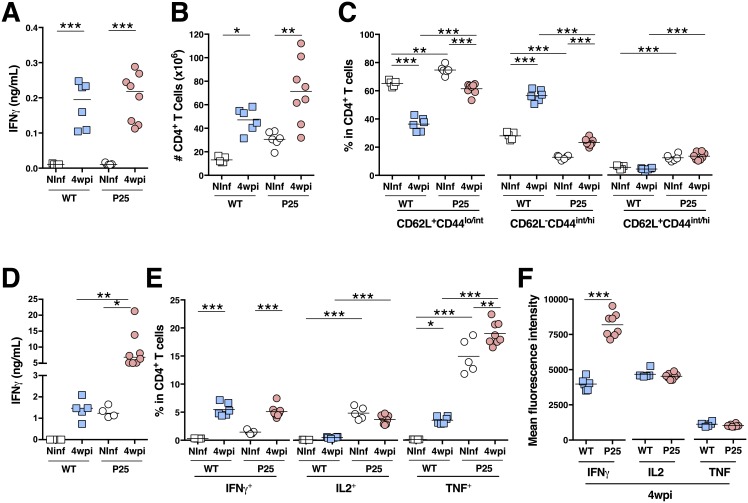
P25 T cells expand and produce protective cytokines throughout *M*. *avium* infection. **A**) IFNγ quantification in the serum from non-infected (Ninf) and from 4 wpi WT and P25 mice. **B**) Enumeration of splenic CD4^+^ T cells. **C**) Percentage of naive (CD62L^+^CD44^lo/int^; left), activated (CD62L^-^CD44^int/hi^; middle) and central memory (CD62L^+^CD44^int/hi^; right) cells among splenic CD4^+^ T cells. **D**) IFNγ quantification in splenocytes’ supernatant upon *ex vivo* stimulation with *M*. *avium* Ag85B_240-254_ peptide. **E**) Percentage of spleen IFNγ (left), IL2 (middle) and TNF^+^ cells among splenic CD4^+^ T cells upon stimulation with *M*. *avium* Ag85B_240-254_ peptide. **F**) Mean fluorescence intensity of IFNγ (left), IL2 (middle) or TNF (left). *p < 0.05, **p < 0.005 and ***p < 0.001 by two-way ANOVA followed by Bonferroni post-hoc tests (A to E) or by Student *t-*test (F). One out of two independent experiments is depicted.

Since IFNγ production by CD4^+^ T cells is a key element in the protective immune response to *M*. *avium* [22,23] the kinetics of CD4^+^ T cell expansion and IFNγ production were analysed. Four weeks upon *M*. *avium* infection, no differences are detected in the serum IFNγ levels and numbers of splenic CD4^+^ T cells between P25 and WT mice (Fig 2A and 2B). Regarding the activation phenotype of the CD4^+^ T cell compartment, non-infected P25 mice show higher percentages of naive (CD62L^+^CD44^lo/int^) and central memory (CD62L^+^CD44^int/hi^) cells, and lower percentages of activated (CD62L^-^CD44^int/hi^) cells in comparison to non-infected WT mice (Fig 2C). These differences between WT and P25 CD4^+^ T cell profiles are preserved or even exacerbated upon infection. In fact, infection leads to more pronounced fold-decrease of naïve T cells in WT mice than in P25 (fold-change of infected to non-infected of 0.56±0.07 *vs*. 0.82±0.05; p<0.0001 by Student *t-*test; Fig 2C).

Upon *ex vivo* stimulation with Ag85_240-254_, splenocytes from infected P25 mice produce ≈3.5-fold more IFNγ than the ones from WT mice (Fig 2D). In addition, the percentage of CD4^+^ T cells producing IL2 or TNF is also higher in P25 in comparison to WT mice (Fig 2E). When testing 4 wpi mice, the percentage of CD4^+^ T cells producing IFNγ is similar for WT and P25 mice, though P25 splenocytes present higher percentages of CD4^+^ T cells producing IL2 or TNF (Fig 2E). Notwithstanding infected WT and P25 mice have similar percentages of CD4^+^ T cells producing IFNγ, P25 CD4^+^ T cells produce more IFNγ than their WT peers, as assessed by the mean fluorescence intensity (MFI; Fig 2F). No differences are detected between both mouse strains for CD4^+^ T cells’ IL2 and TNF MFI. Altogether, these data show that P25 mice control infection similarly to WT, although with a distinct CD4^+^ T cell activation and cytokine producing profile. The higher production of pro-inflammatory cytokines in P25 mice lead us to question if a potentially excessive immune response would take place leading to immunopathology.

### *M*. *avium*-infected P25 mice present no signs of increased immunopathology in comparison to WT mice

**Fig 3:**
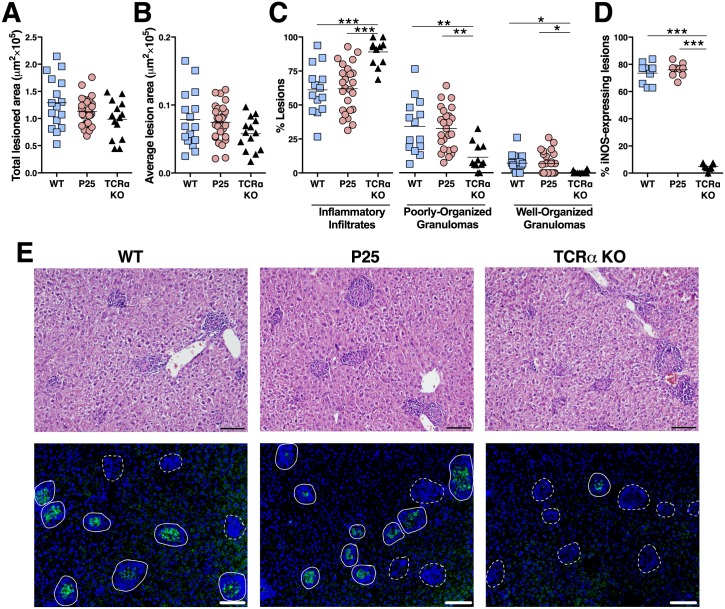
No signs of immunopathology are detected in *M*. *avium* chronic infected P25 mice. **A**) Assessment of the total lesioned area. **B**) Quantification of the average lesion size. **C**) Classification and percentage of each lesion type. **D**) Percentage of liver iNOS^+^ lesions. *p < 0.05, **p < 0.005 and ***p < 0.001 by one-way ANOVA test followed by Bonferroni post-hoc tests. A pool from three independent experiments is depicted. **E**) Representative histological liver sections stained by hematoxylin and eosin (top) or stained for iNOS (bottom); in the later, iNOS^+^ lesions (agglomerates of DAPI^+^ nuclei accompanied by iNOS stain; filled outlines) and iNOS^-^ lesions (agglomerates of DAPI^+^ nuclei without iNOS stain; dashed outlines) are depicted. All assessments were performed on liver sections of WT, P25 and TCR*α* KO mice at 16 wpi. Scale bar, 100 μm.

As splenocytes from infected P25 mice are more prone to produce IFNγ and TNF compared to WT, we questioned if control of bacterial growth in P25 mice was accompanied by increased immunopathology. Excessive leukocyte infiltration and altered granuloma profile have been shown in mouse models of excessive immune response [24–26]. The total area with inflammatory infiltrates and the average lesion size were measured in WT, P25 and TCRα KO mice at 16 wpi. No differences are detected for both measurements (Fig 3A and 3B). Additionally, lesions’ structure was categorized as inflammatory infiltrates, well- or poorly-organized granulomas [27]; P25 and WT mice present no differences in between them and both differ significantly from infected TCRα KO mice, which are practically deprived of well-organized granulomas (Fig 3C and 3E top).

Cytokine production, particularly IFNγ and TNF, are key for Mϕ activation and the initiation of bactericidal mechanisms, such as the one mediated by the iNOS [22,28–31]. Although iNOS KO mice are significantly more efficient in clearing *M*. *avium* infection in comparison to WT mice [32] this enzyme is still a suitable marker for Mϕ activation during *M*. *avium* infection. A similar percentage of iNOS^+^ lesions are present in WT and P25 mice and, once again, both groups differ significantly from TCRα KO mice, which present a smaller frequency of iNOS^+^ lesions (Fig 3D and 3E bottom). Altogether, these data suggest no increased immunopathology in infected P25 mice in comparison to WT mice.

### TCR transgenic CD4^+^ T cells are the ones preferentially activated during the immune response to *M*. *avium* in P25 mice

**Fig 4:**
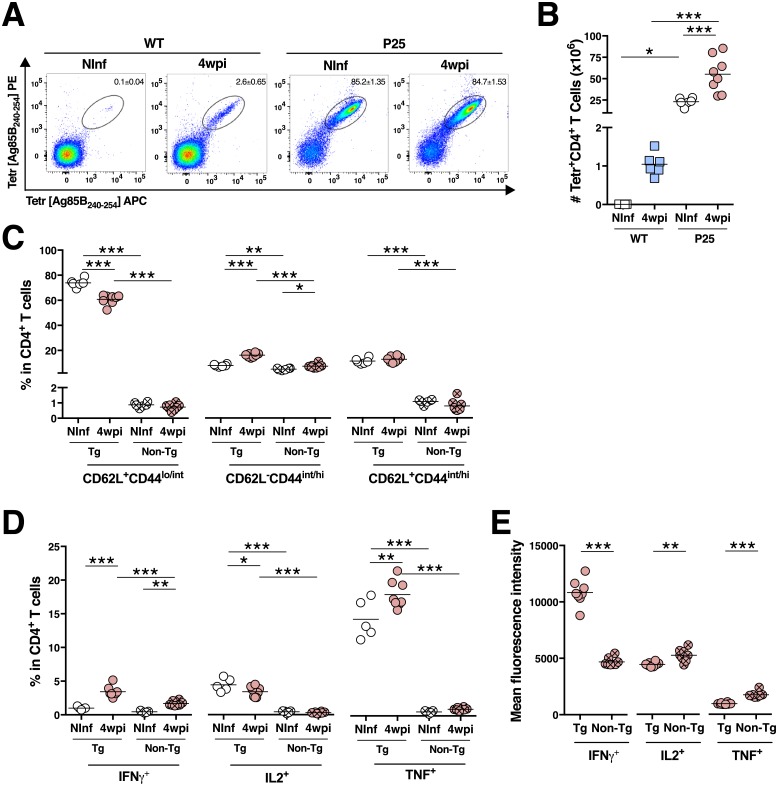
Upon *M*. *avium* infection of P25 mice, TCR transgenic CD4^+^ T cells are preferably more activated then their non-transgenic peers. **A**) Representative FACS plot of Ag85B_240-254_-tetramer^+^ cells gated on CD4^+^ T cells. **B**) Enumeration of Ag85B_240-254_-tetramer^+^ CD4^+^ T cells in the spleen of non-infected (Ninf) and of 4 wpi WT and P25 mice. **C**) Percentage of naive (CD62L^+^CD44^lo/int^; left), activated (CD62L^-^CD44^int/hi^; middle) and central memory (CD62L^+^CD44^int/hi^; right) cells which are TCR transgenic (Tg; identified as Vα_Mix_^-^Vβ11^+^) or non-transgenic (Non-Tg; identified as all that are not Vα_Mix_^-^Vβ11^+^) among CD4^+^ T cells from spleens from non-infected or from 4wpi P25 mice. **D**) Percentage of IFNγ^+^ (left), IL2^+^ (middle) and TNF^+^ cells (left) upon *ex vivo* stimulation with *M*. *avium* Ag85B_240-254_, among P25 transgenic or non-transgenic CD4^+^ T cells. **E**) Mean fluorescence intensity of IFNγ (left), IL2 (middle) and TNF (left), among cytokine-positive transgenic or non-transgenic CD4^+^ T cells. *p < 0.05, **p < 0.005 and ***p < 0.001 by two-way ANOVA followed by Bonferroni post-hoc tests (B to D) or by Student *t-*test (E). One out of three independent experiments is depicted.

The P25 mice used in this study are in a RAG-sufficient background, enabling the differentiation of a minor T cell population expressing non-transgenic TCRs. For that reason, these mice have cells specific for antigens other than Ag85_240-254_. We investigated to what extent the CD4^+^ T cells non-specific for Ag85_240-254_ would play a relevant role on the immune response of P25 mice to *M*. *avium* infection. Ag85_240-254_-loaded I-A^b^ tetramers were used to identify the Ag85_240-254_-specific CD4^+^ T cells, revealing that this T cell population is very scarce in non-infected WT mice and very abundant in P25 mice (over 85%; Fig 4A). The number of these Ag85-specific CD4^+^ T cells expands during infection in both mouse strains (Fig 4B). Because the tetramer staining protocol coupled to *ex vivo* stimulation and intracellular staining protocol is technically problematic, specific antibodies for the TCR chains were used. The P25 transgenic TCR is composed by a Vα5 and a Vβ11 TCR chains; as there are no specific antibodies for the TCR Vα5 chain, identification of the transgenic TCR was done using anti-Vβ11 plus a mixture containing all the commercially available antibodies directed to other TCR Vα chains (Vα_Mix_, *i*.*e*. the Vα2, 3.2, 8.3, 11.1 and 11.2 chains). P25 transgenic cells were defined as Vα_Mix_^-^Vβ11^+^, and the non-transgenic defined as all the CD4^+^ T cells that are not Vα_Mix_^-^Vβ11^+^ (S1A Fig). P25 transgenic cells constitute around 85% of the CD4^+^ T cells (S3A Fig) and the vast majority of those cells stain with Ag85_240-254_-tetramers (S3B and S3C Fig). A small percentage of Vα_Mix_^+^Vβ11^+^ CD4^+^ T cells also binds the Ag85_240-254_ tetramer however this population represents only a minor population of the total CD4^+^ T cells (0.45±0.04%; S3B Fig). These data indicate that the use of the Vβ11 antibody together with the Vα_Mix_ antibodies enables the identification of most of the Ag85-specific cells.

Similar to the total CD4^+^ T cell compartment within P25 mice upon infection, the percentage of naive P25 transgenic CD4^+^ T cells decreases (Fig 4C left), while the activated population increases (Fig 4C middle). Although some non-transgenic CD4^+^ T cells also acquire an activated phenotype upon infection, the fold-change is higher in the P25 transgenic cells in comparison to the non-transgenic (2.05±0.21 *vs*. 1.48±0.34, *p*<0.0001 by Student *t-*test). Regarding cytokine production, infected P25 transgenic CD4^+^ T cells are enriched with IFNγ^+^ and TNF^+^ CD4^+^ T cells when compared with non-infected peers (Fig 4D). Despite non-transgenic cells become also enriched with IFNγ^+^ cells upon infection, a higher percentage of P25 transgenic than non-transgenic CD4^+^ T cells producing cytokines is detected (Fig 4D). Additionally, P25 transgenic cells also present higher IFNγ MFIs than non-transgenic CD4^+^ T cells, though the last have higher TNF and IL2 MFIs (Fig 4E).

## Discussion

In this study, we used P25 mice in a RAG-sufficient background to investigate the immune response to *M*. *avium* mediated by a repertoire clearly, but not exclusively, dominated by Ag85-specific T cells. We show that an immunodominant T cell response to Ag85 controls the bacterial burden to the same level as a WT diverse T cell repertoire. Although upon infection P25 mice present a greater increase in the proportion of activated CD4^+^ T cells, there are no signs of *in vivo* higher production of IFNγ or of immunopathology in comparison to WT mice. Curiously, when stimulated *ex vivo*, cells from P25 mice produce more IFNγ, IL2 and TNF. Although P25 mice harbour non-transgenic CD4^+^ T cells, activation and immune response is clearly dominated by the P25 TCR transgenic CD4^+^ T cells.

Data available on the Ag85-directed immune response in the context of *M*. *tuberculosis* infection advocate this peptide as an early-secreted antigen, as its expression peaks around 20 dpi and because Ag85-specific CD4^+^ T cells fail to proliferate when transferred to *M*. *tuberculosis* infected mice with over 4 weeks of infection. To our knowledge, only one report is available describing the bacterial burden of P25 mice infected by *M*. *tuberculosis*; this evaluation was performed at 4 wpi and no information exists for longer periods of infection [17]. Here we show that in the *M*. *avium* infection model, protection conferred by P25 transgenic T cells is sustained throughout the 20 weeks of infection evaluated. The fact that we observe in the P25 mouse model a TCR repertoire sufficient to sustain an immune response to *M*. *avium* might be related to the fact that splenocytes from *M*. *avium* infected mice, when stimulated with antigenic fractions from culture filtrate, envelope or cytosol, produce the highest levels of INFγ in response to the Ag85-containing fractions [8]. These data suggest that the immune response to Ag85 might be preponderant for the control of the *M*. *avium* bacterial growth.

A recent report by Moguche and colleagues has shown that in the context of *M*. *tuberculosis* infection, the immune response to Ag85 was arrested due to the reduced antigen availability during persistent infection; these Ag85-specific CD4^+^ T cells contracted in number and exhibited a less differentiated phenotype (similar to memory cells) with the ability to produce multiple cytokines [14]. Our data suggests that the immune response in *M*. *avium*-infected P25 mice is supported by the continuous expression of Ag85 by the bacilli, as we are able to clearly detect Ag85-staining in most *M*. *avium* infected cells from 20 wpi mice. Nevertheless, we did not investigate whether the expression of this antigen is altered from the acute to the chronic phase of the *M*. *avium* infection, as described for *M*. *tuberculosis* [12].

We observe a similar fold-increase for both IFNγ serum levels and CD4^+^ T cells numbers in both WT and P25 infected mice when compared with their non-infected peers (15.56±5.60 *vs*. 17.74±6.12, *p-*value = 0.5078; 3.24±0.91 *vs*. 3.73±1.42, *p-*value = 0.4524, respectively. Student *t-*tests were performed). These results are quite interesting since infected WT and P25 differ significantly in the frequency of activated CD4^+^ T cells present during infection, even though progressing similarly when compared with their non-infected peers. We hypothesize that infection does not generate sufficient Ag85 protein levels to stimulate all P25 CD4^+^ T cells to their full potential. Supporting this idea is the observed massive IFNγ levels seen after *ex vivo* stimulation of splenocytes from infected P25 mice with *M*. *avium*-Ag85_240-254_ peptide (3.5-fold more IFNγ is produced by infected P25 mice in comparison to WT), a condition where more cognate-antigen is available. In line with this, and despite the percentage of IFNγ-producing CD4^+^ T cells in P25 and WT mice being similar, these cells have higher IFNγ MFI than the ones from WT mice, which may also suggest an enhanced IFNγ-producing phenotype. However, the existence of host regulatory mechanisms that prevent and protect from an excessive immune response can not be excluded. In fact, no increase in liver immunopathology is detected in P25 mice, in comparison with WT mice.

As previously referred, the P25 mice used in this study are in a RAG-sufficient background. In this condition, the presence of CD4^+^ T cells that are not specific for Ag85 (which compose less than 15% of the CD4^+^ T cell compartment) could be eliciting most of the immune response to the bacteria. Against this, we observe an expansion of Ag85-specific CD4^+^ T cells with infection, alterations on the T cell activation profile that are more exuberant in the transgenic over the non-transgenic cells and more IFNγ^+^ and TNF^+^ CD4^+^ T cells in the transgenic compartment. All together, these results suggest that the immune response in the P25 mice is mostly driven by P25 transgenic CD4^+^ T cells, and that this highly skewed repertoire is as protective as a diverse one. Extremely skewed repertoires have been shown to be protective in the context of several viral infections. However, in the case of viruses highly prone to mutations this restricted immune response to immunodominant epitopes might be detrimental as it becomes evaded upon mutation [33]. Both in humans and in mouse models, a clonal expansion, mostly of CD8^+^ T cells, during *M*. *tuberculosis* infection arises and, in the specific case of C57BL/6 mice, 30 to 50% of the CD8^+^ T cells recognise a single epitope upon infection [34–36]; prominent TCR clonal expansions have been associated with more severe tuberculosis cases [37,38]. However, these reports could not clearly demonstrate whether the strongly skewed repertoire was the cause of disease severity or vice-versa, a subject that remains to be explored.

Collectively, our results suggest that an immune response dominated towards Ag85 is sufficient to promote long-term protection to *M*. *avium*. Importantly, despite the prevalent response mediated by P25 transgenic T cells, no increased immunopathology is detected. This information may be of relevance for the antigen-based vaccine strategies.

## Supporting information

S1 Fig Flow cytometry gating strategies. **A**) Representative gating strategy used to identify naive (CD62L^+^CD44^lo/int^), activated (CD62L^-^CD44^int/hi^) and central memory (CD62L^+^CD44^int/hi^) CD4^+^ T cells. **B**) Representative gating strategy used to identify IFN, IL2 and TNF producing CD4^+^ T cells.
(EPS)

S2 Fig Unlike P25 mice, Limited mice, which also harbor a restricted TCR repertoire, are unable to control *Mycobacterium avium* infection. Quantification of spleen (left) and liver (right) colony forming units (CFUs) from WT (Exp#3), P25 (Exp#1), TCRα KO (Exp#2 and 3) and Limited mice (Exp#2 and 3) with 24 to 27 weeks post-infection (dpi). No significant differences were observed on Exp#2 by Student *t*-test. ***p < 0.001 by one-way ANOVA test and followed by Bonferroni post-hoc tests (Exp#3). Each symbol represents one mouse and the line the mean.
(JPG)

S3 Fig TCR staining as an adequate surrogate marker for transgenic CD4^+^ T cells in P25 mice. **A)** Representative FACS plot of the different CD4^+^ T cells sub- populations according to the expression of Vα_Mix_(2, 3, 8 and 11) and Vβ11 (left). **B)** Percentage of Ag85B_240-254_-specific (Tetramer^+^) CD4^+^ T cells among each of four sub-populations based on Vα_Mix_ and Vβ11 staining. **C)** Percentage of Tetramer^+^ cells on each of four sub-populations based on Vα_Mix_ and Vβ11 staining within CD4^+^ T cells. ***p < 0.001 by one-way ANOVA test followed by Bonferroni post-hoc test. Each symbol represents one mouse, the line the mean; data representative of two independent experiments. Tetramer stain did not compromise TCR stain, since single (tetramer stain only) or multiple (tetramer stain followed by Vβ11 and Vα_Mix_ stain) rendered similar Vβ11^+^Vα^-^ percentages (84.3±2.9 *vs*. 85.1±2.1, *p* = 0.8945).
(EPS)
