# Supplementary figures and images for: Ag85-focused T-cell immune response controls Mycobacterium avium chronic infection
Source: PLoS One. 2018 Mar 2;13(3):e0193596. doi: 10.1371/journal.pone.0193596 (PMC5834192; doi:10.1371/journal.pone.0193596)

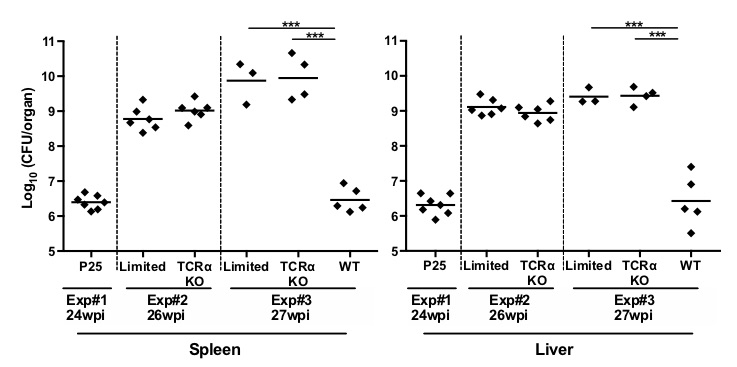

Supplement: S2 Fig — Quantification of spleen (left) and liver (right) colony forming units (CFUs) from WT (Exp#3), P25 (Exp#1), TCRα KO (Exp#2 and 3) and Limited mice (Exp#2 and 3) with 24 to 27 weeks post-infection (dpi). No significant differences were observed on Exp#2 by Student t-test. ***p < 0.001 by one-way ANOVA test and followed by Bonferroni post-hoc tests (Exp#3). Each symbol represents one mouse and the line the mean. (JPG) [file pone.0193596.s002.jpg]
